# Supplementary material for: Activation mechanism of PINK1
Source: Nature. 2021 Dec 21;602(7896):328–35. doi: 10.1038/s41586-021-04340-2 (PMC8828467; doi:10.1038/s41586-021-04340-2)
Supplement: Supplementary file 1 — Supplementary Fig. 1: uncropped gels and blots. Supplementary Fig. 2: repeats of Fig. 5e, used for quantification in Fig. 5f. [file 41586_2021_4340_MOESM1_ESM.pdf]

nature portfolio

https://doi.org/10.1038/s41586-021-04340-2

Supplementary information

Activation mechanism of PINK1

In the format provided by the authors and unedited

Supplementary Figure 1.

Supplementary Figure 1. Uncropped gels and blots for all Figures.
Areas outlined by a disrupted box were used to assemble Figure panels. Molecular weight markers indicate size in kDa. Phos-tag gels in which proteins run according to phosphorylation status and not size, are indicated.

Figure 3g

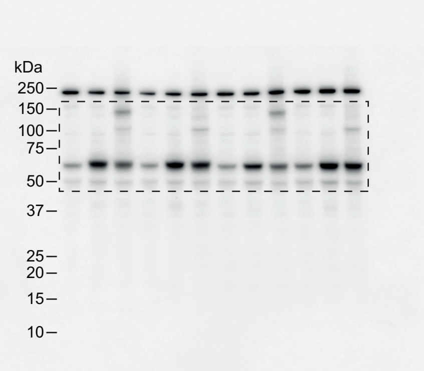

anti-PINK1 (non-reducing)

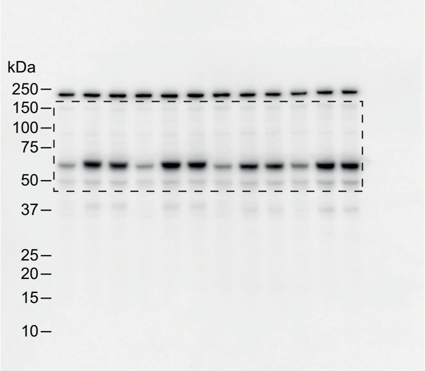

anti-PINK1

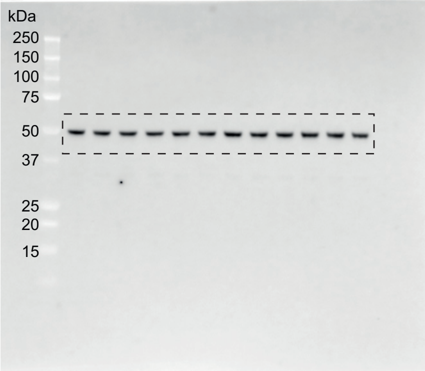

anti-tubulin

Extended Data Figure 5f

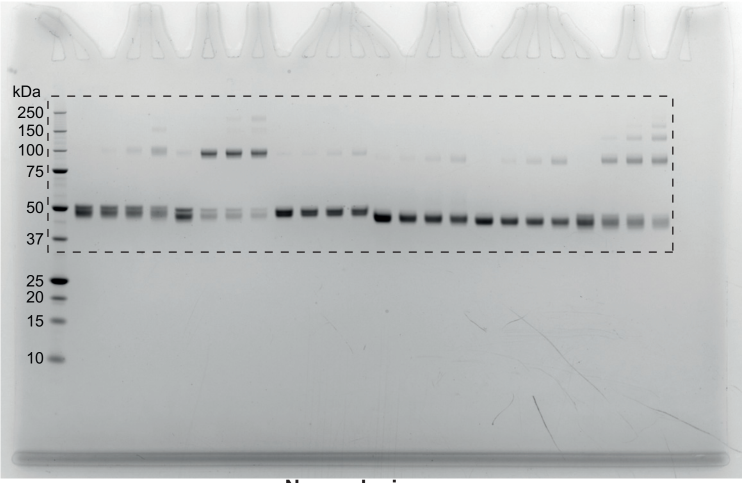

Non-reducing

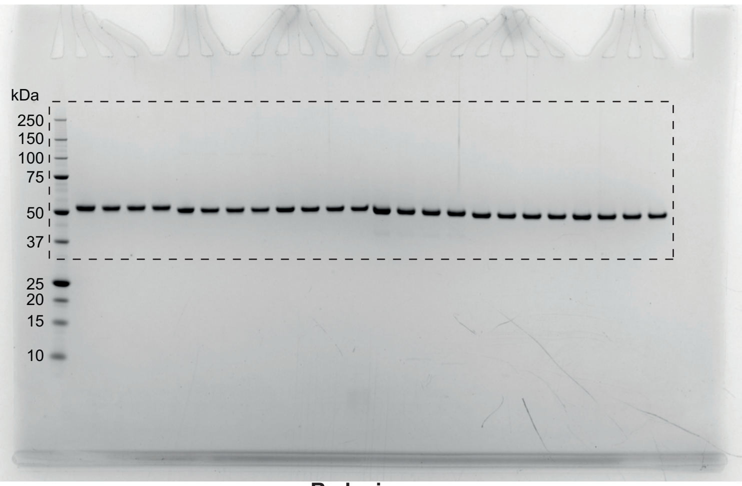

Reducing

Extended Data Figure 5h

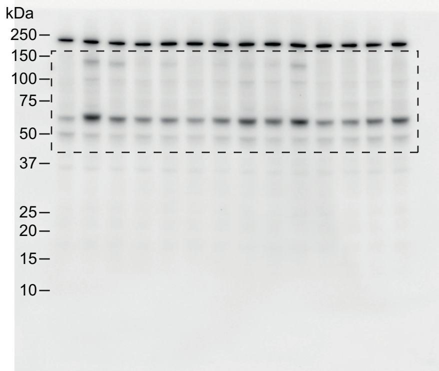

anti-PINK1 (non-reducing)

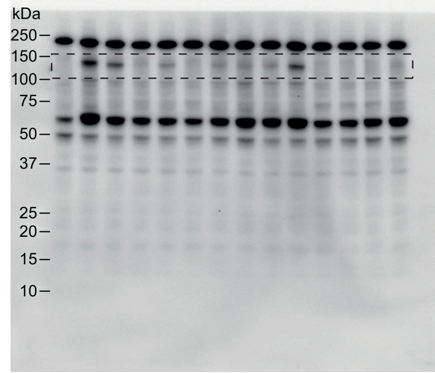

anti-PINK1 (non-reducing, high exposure)

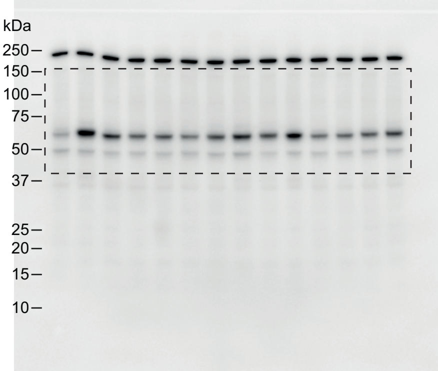

anti-PINK1

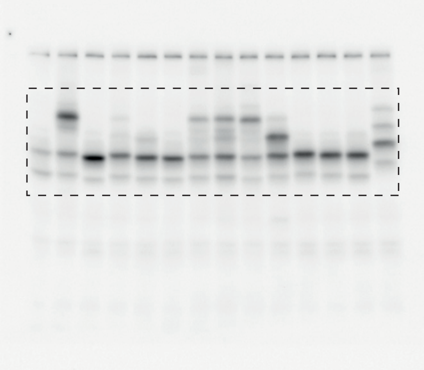

anti-PINK1 (Phos-tag)

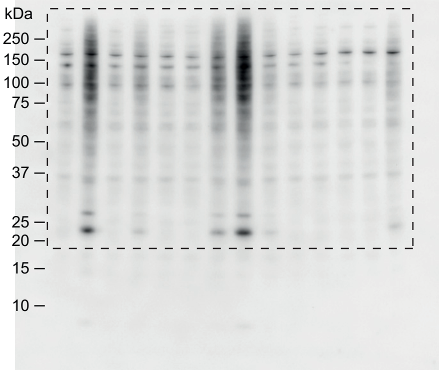

anti-pSer65-Ub

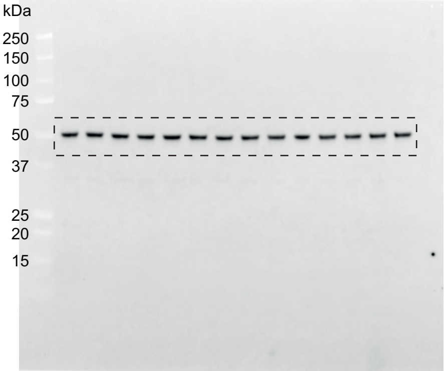

anti-tubulin

Extended Data Figure 6a

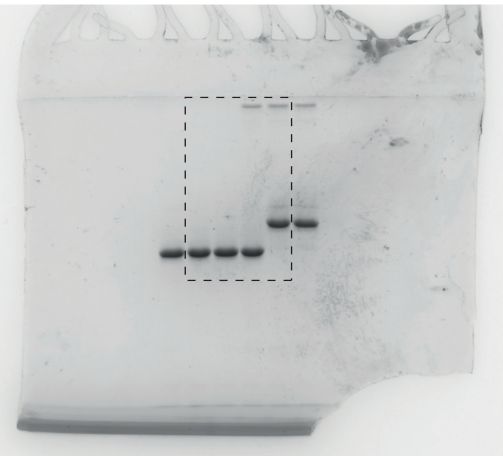

(Phos-tag)

Extended Data Figure 6b

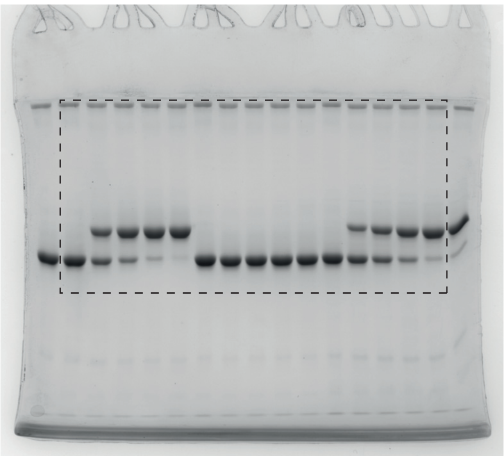

(Phos-tag)

Extended Data Figure 6d

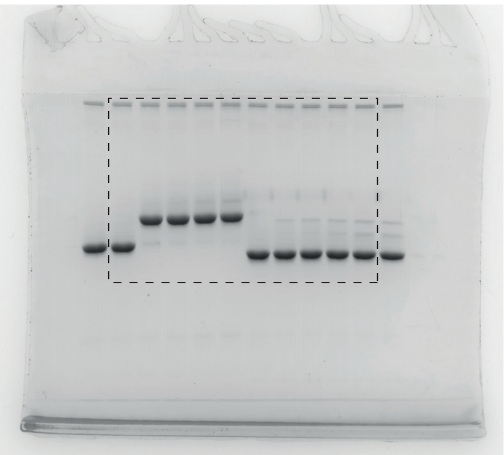

(Phos-tag)

Extended Data Figure 6e

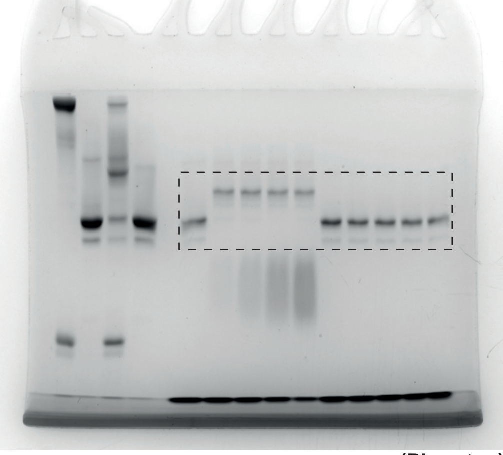

7.5% acrylamide (Phos-tag)

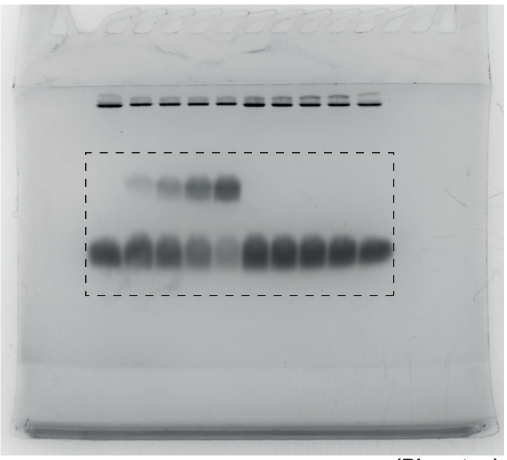

17.5% acrylamide (Phos-tag)

Extended Data Figure 6h

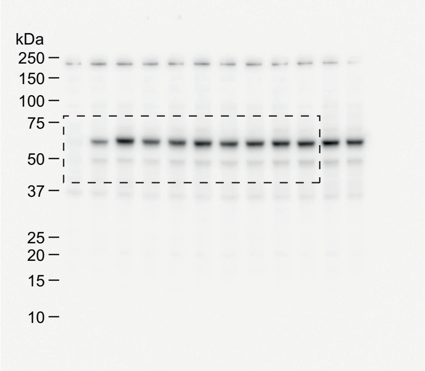

anti-PINK1

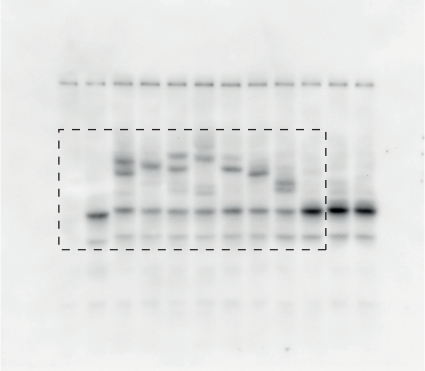

anti-PINK1 (Phos-tag)

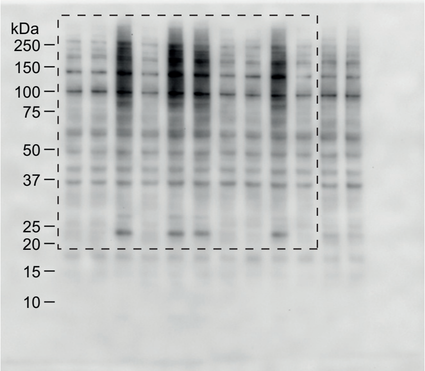

anti-pSer65-Ub

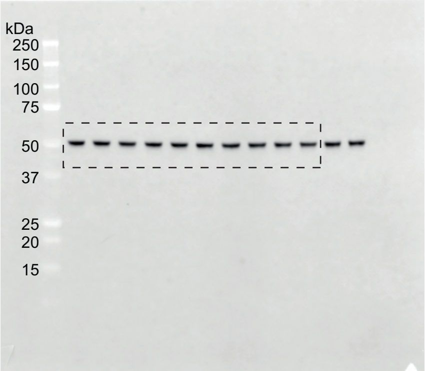

anti-tubulin

Extended Data Figure 6i

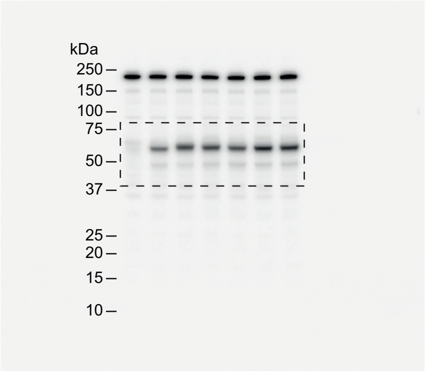

anti-PINK1

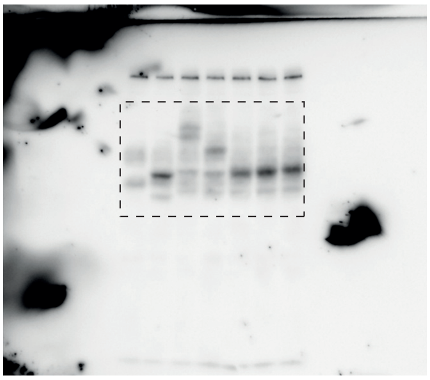

anti-PINK1 (Phos-tag)

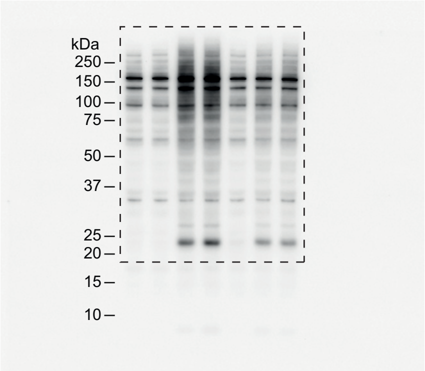

anti-pSer65-Ub

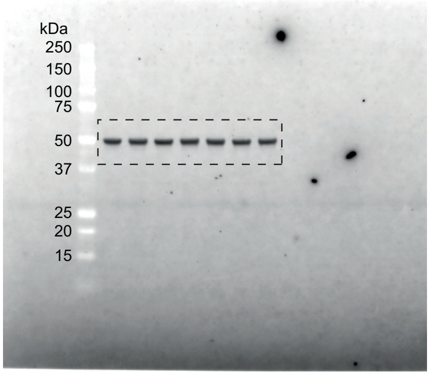

anti-tubulin

Figure 4c

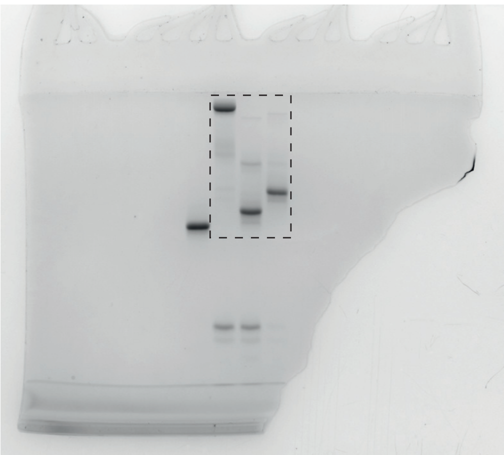

(Phos-tag)

Extended Data Figure 7a

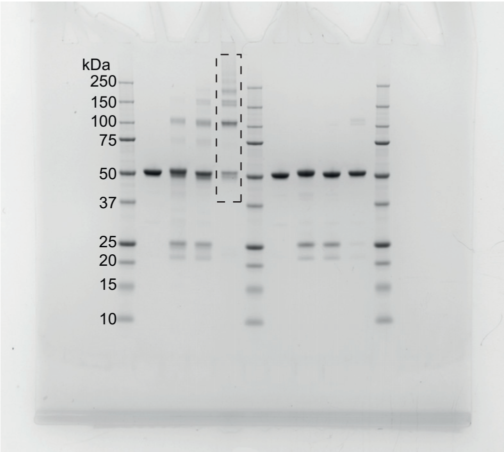

Extended Data Figure 8d

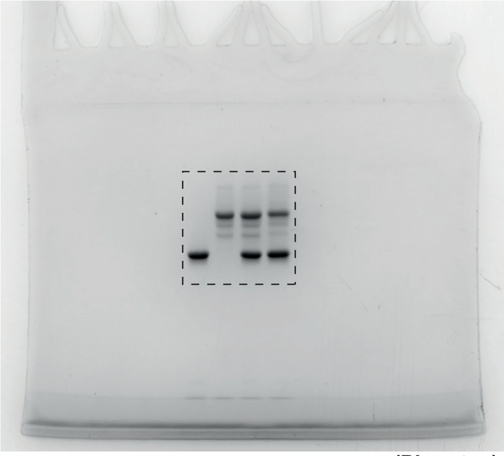

(Phos-tag)

Figure 5b

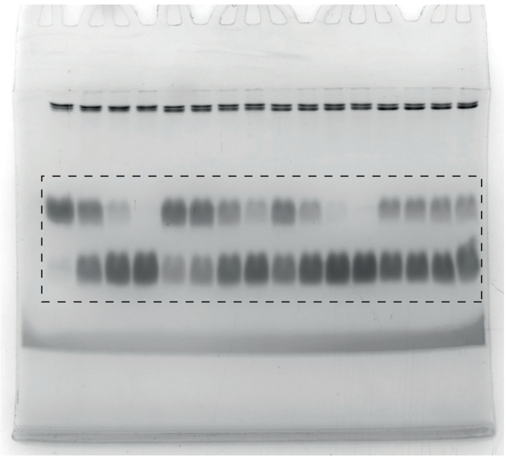

(Phos-tag)

Extended Data Figure 9b

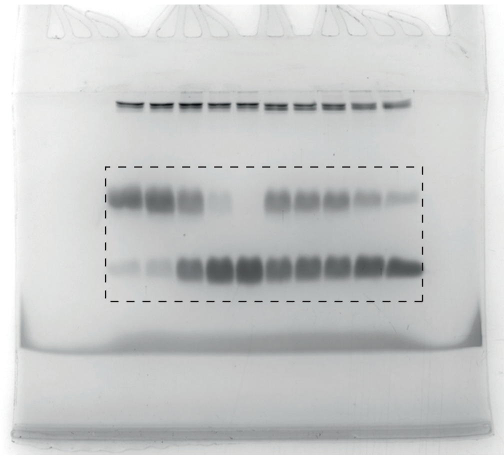

(Phos-tag)

Extended Data Figure 9c

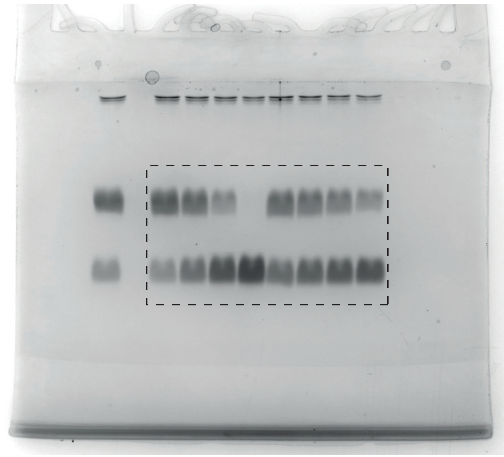

(Phos-tag)

Extended Data Figure 9d

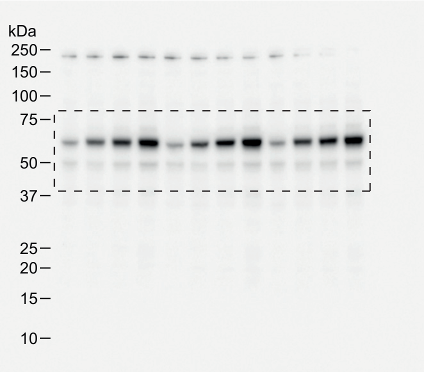

anti-PINK1

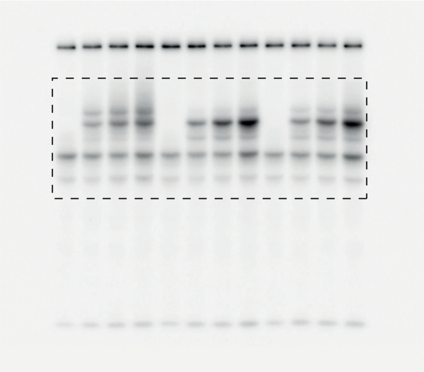

anti-PINK1 (Phos-tag)

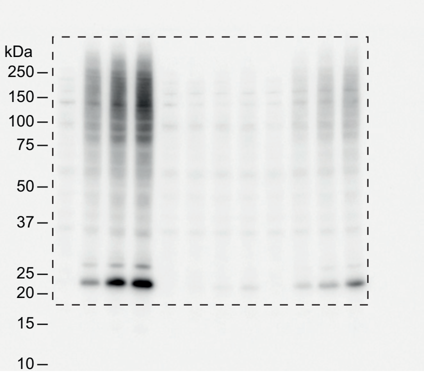

anti-pSer65-Ub

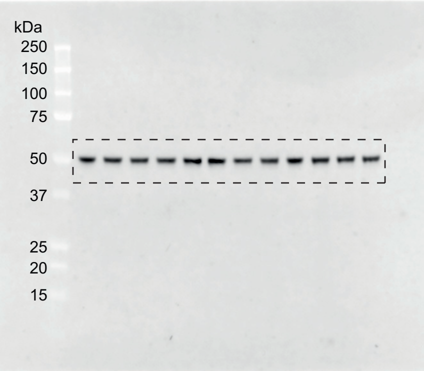

anti-tubulin

Extended Data Figure 9e

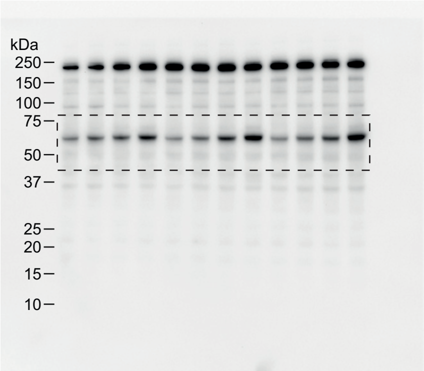

anti-PINK1

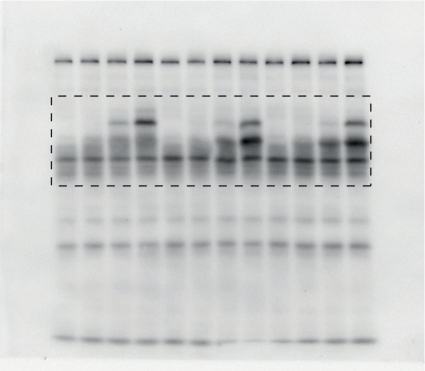

anti-PINK1 (Phos-tag)

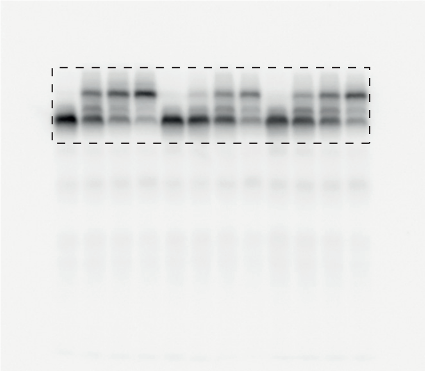

anti-Parkin (Phos-tag)

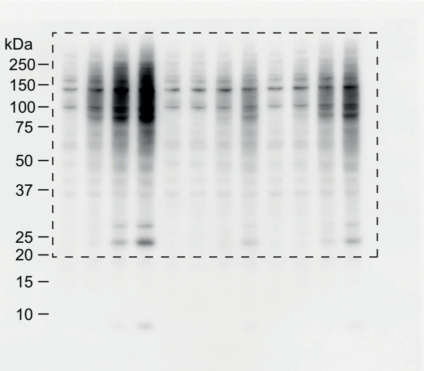

anti-pSer65-Ub

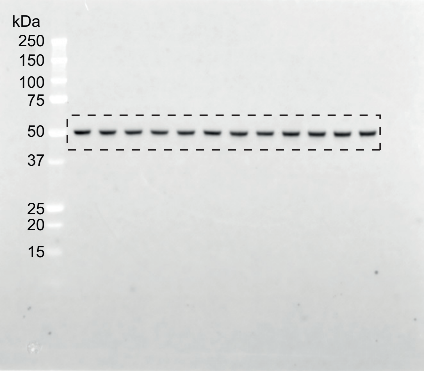

anti-tubulin

Extended Data Figure 9h

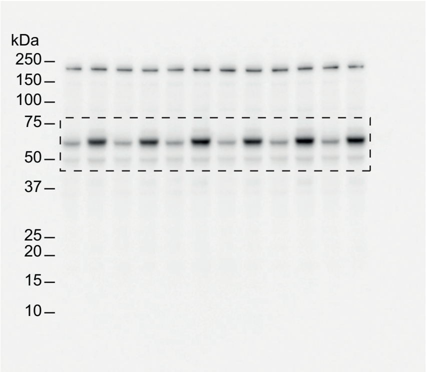

anti-PINK1

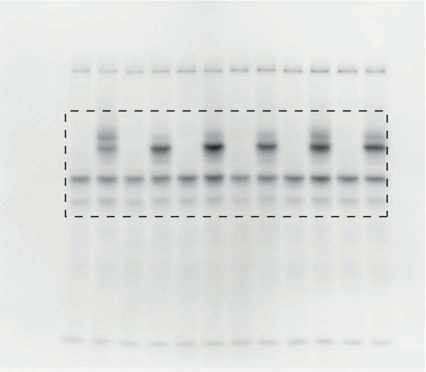

anti-PINK1 (Phos-tag)

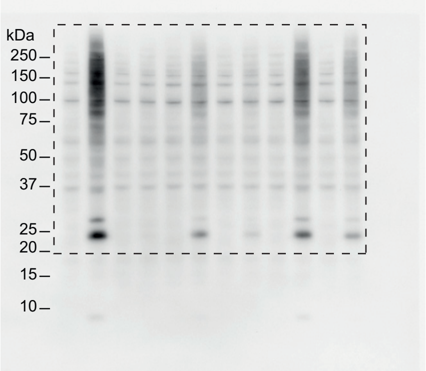

anti-pSer65-Ub

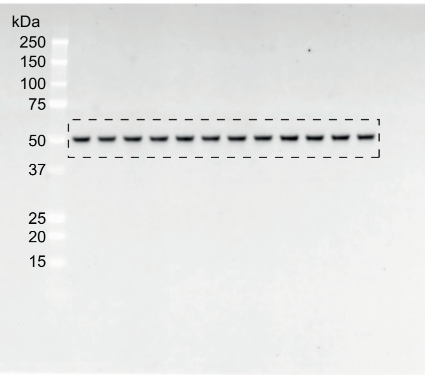

anti-tubulin

Figure 5e

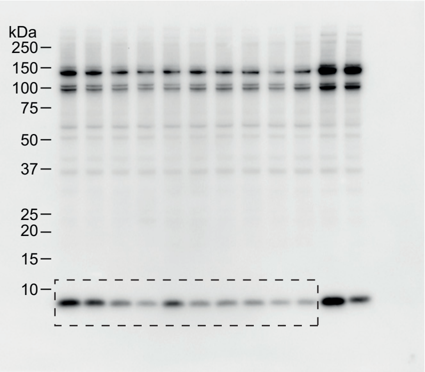

anti-pSer65-Ub

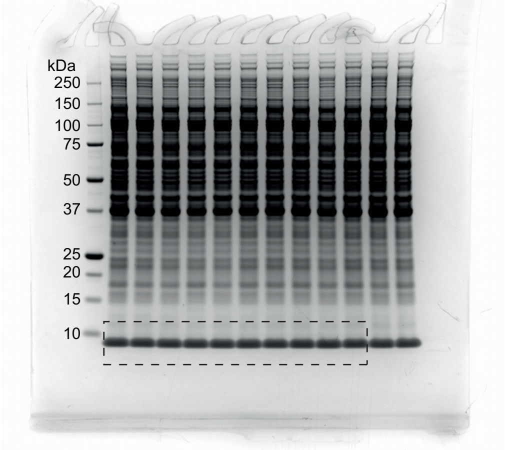

Coomassie

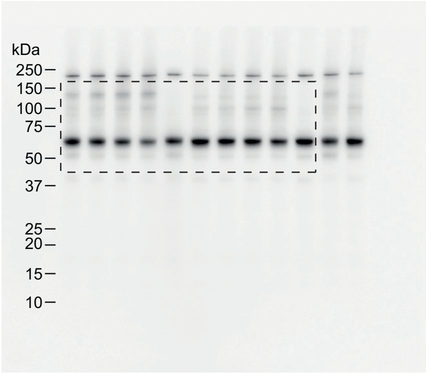

anti-PINK1 (non-reducing)

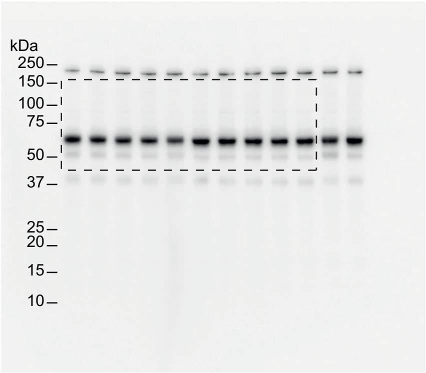

anti-PINK1

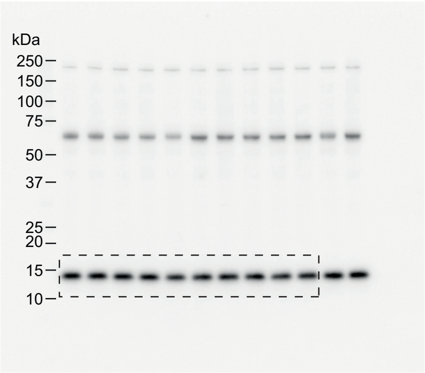

anti-TOM20

Repeats of Figure 5e for quantification in Figure 5f, displayed in Supplementary Figure 2

Repeat 1

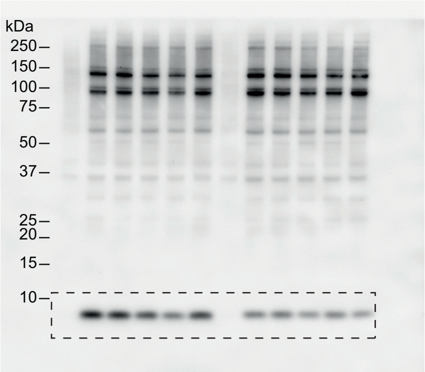

anti-pSer65-Ub

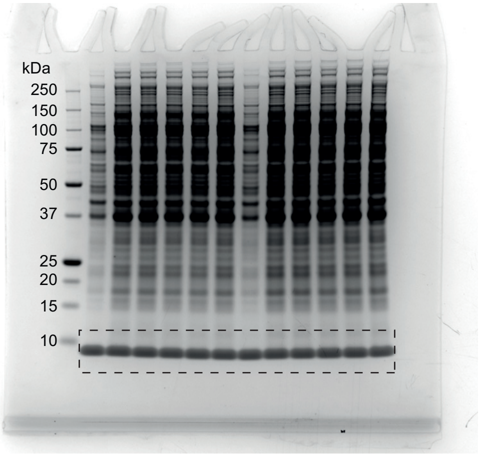

Coomassie

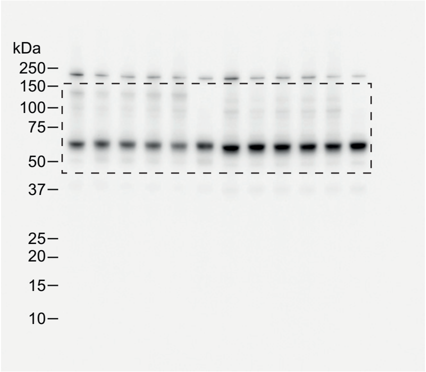

anti-PINK1 (non-reducing)

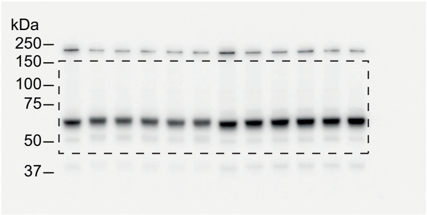

anti-PINK1

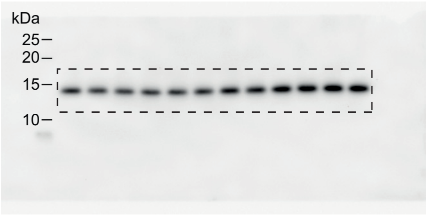

anti-TOM20

Repeat 2

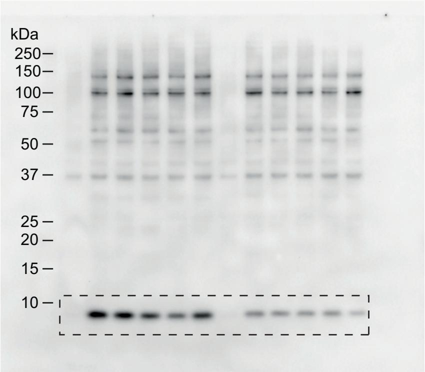

anti-pSer65-Ub

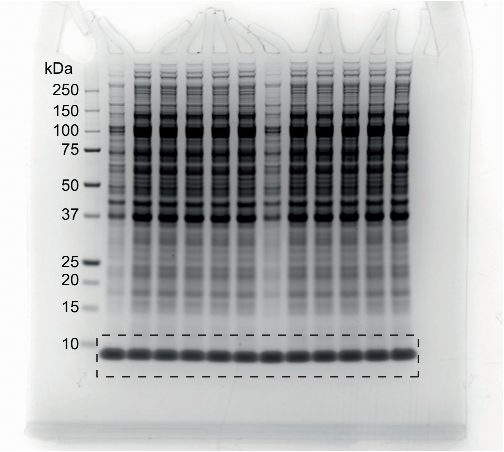

Coomassie

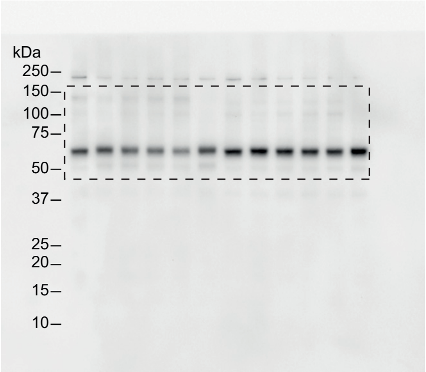

anti-PINK1 (non-reducing)

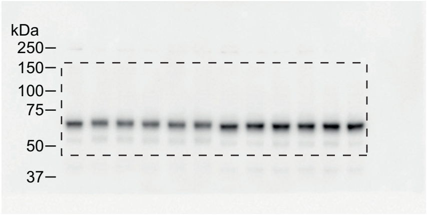

anti-PINK1

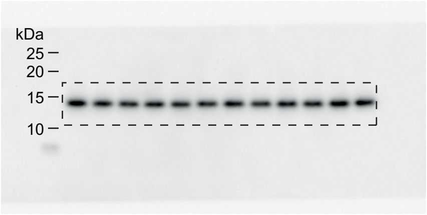

anti-TOM20

Supplementary Figure 2

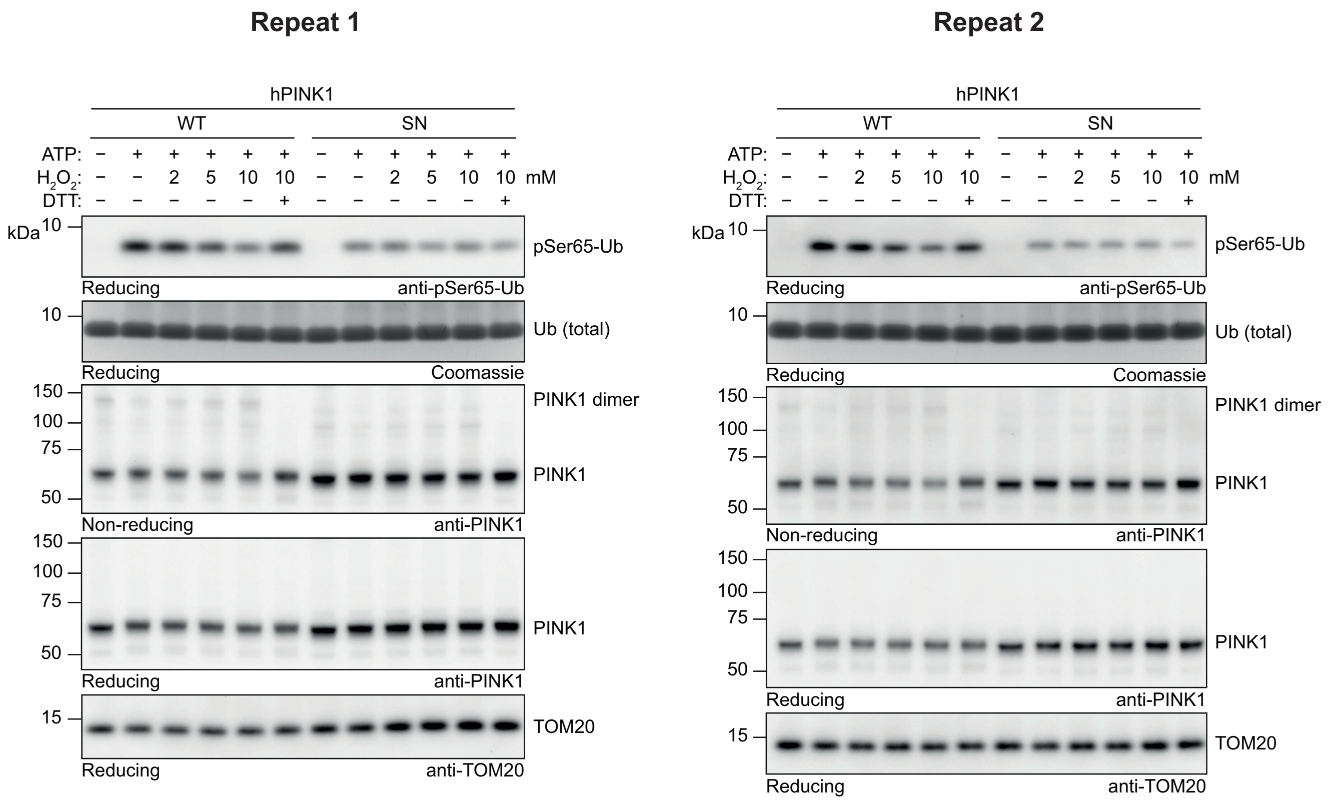

Supplementary Figure 2. Repeats of Figure 5e, used for quantification in Figure 5f. Experiments for **Figure 5e** were performed in three independent, biological repeats according to the workflow depicted in **Figure 5d** (see **Methods**). The repeat experiments are shown as in **Figure 5e** (uncropped blots part of **Supplementary Figure 1**), phospho-Ser65 ubiquitin bands were quantified, and individual data points plotted in **Figure 5f**.
