# Supplementary material for: Activation mechanism of PINK1
Source: Nature. 2021 Dec 21;602(7896):328–35. doi: 10.1038/s41586-021-04340-2 (PMC8828467; doi:10.1038/s41586-021-04340-2)
Supplement: Supplementary file 2 — Reporting Summary [file 41586_2021_4340_MOESM2_ESM.pdf]

nature research

Corresponding author(s): David Komander

Last updated by author(s): 13/12/21

Reporting Summary

Nature Research wishes to improve the reproducibility of the work that we publish. This form provides structure for consistency and transparency in reporting. For further information on Nature Research policies, see our Editorial Policies and the Editorial Policy Checklist.

Statistics

For all statistical analyses, confirm that the following items are present in the figure legend, table legend, main text, or Methods section.

n/a	Confirmed	
☐	☒	The exact sample size (*n*) for each experimental group/condition, given as a discrete number and unit of measurement
☐	☒	A statement on whether measurements were taken from distinct samples or whether the same sample was measured repeatedly
☐	☒	The statistical test(s) used AND whether they are one- or two-sided<br>*Only common tests should be described solely by name; describe more complex techniques in the Methods section.*
☒	☐	A description of all covariates tested
☒	☐	A description of any assumptions or corrections, such as tests of normality and adjustment for multiple comparisons
☒	☐	A full description of the statistical parameters including central tendency (e.g. means) or other basic estimates (e.g. regression coefficient) AND variation (e.g. standard deviation) or associated estimates of uncertainty (e.g. confidence intervals)
☐	☒	For null hypothesis testing, the test statistic (e.g. *F*, *t*, *r*) with confidence intervals, effect sizes, degrees of freedom and *P* value noted<br>*Give P values as exact values whenever suitable.*
☒	☐	For Bayesian analysis, information on the choice of priors and Markov chain Monte Carlo settings
☒	☐	For hierarchical and complex designs, identification of the appropriate level for tests and full reporting of outcomes
☒	☐	Estimates of effect sizes (e.g. Cohen's *d*, Pearson's *r*), indicating how they were calculated

Our web collection on statistics for biologists contains articles on many of the points above.

Software and code

Policy information about availability of computer code

Data collection	ÄKTA Pure (Cytiva), DAWN light scattering detector (Wyatt Technology), Optilab T-rEX Refractive Index Detector (Wyatt Technology), Rotor-Gene Q (Qiagen), timsTOFII Pro Mass Spectrometer (Bruker), ChemiDoc (Bio-Rad), Talos L120C TEM (Thermo Scientific), Talos Arctica TEM (Thermo Scientific); Titan Krios G4 TEM (Thermo Scientific); Lattice Light Sheet (LLS) 7 (ZEISS - pre-serial)
Data analysis	Crystallography: XDSme (0.6.5.2), CCP4i (7.0.001), POINTLESS (1.10.18), AIMLESS (0.5.21), Phenix (1.19.2-4158-000), Phaser (2.8.3), MolProbity (4.5.1), Coot (0.9), UCSF ChimeraX (1.1.1)<br>Cryo-EM: EPU software (2.9), Relion (3.1), cryoSPARC (3.2.0), UCSF Chimera (1.14), UCSF ChimeraX (1.1.1), Coot (0.9), EMAN (2.3.1)<br>Biochemistry: GraphPad Prism (9.0.0), Image Lab (Bio-Rad, 6.1), ASTRA software (Wyatt technology, 7.3.1.9), Rotor-Gene Q Series Software (2.3.1), MaxQuant (1.6.17.0), Skyline Daily (21.1.1.198), UNICORN 7 (Cytiva, 7.5 and 7.6)<br>Imaging: ZEN (ZEISS, 3.5), Fiji (ImageJ, 1.53k), Matlab (R2019b)

For manuscripts utilizing custom algorithms or software that are central to the research but not yet described in published literature, software must be made available to editors and reviewers. We strongly encourage code deposition in a community repository (e.g. GitHub). See the Nature Research guidelines for submitting code & software for further information.

Data

Policy information about availability of data

All manuscripts must include a data availability statement. This statement should provide the following information, where applicable:

- Accession codes, unique identifiers, or web links for publicly available datasets
- A list of figures that have associated raw data
- A description of any restrictions on data availability

Coordinates and crystallographic structure factors for PhPINK1 D334A have been deposited with the protein data bank under accession codes 7T3X, and EM models and maps under accession codes 7T4M (PhPINK1 D357A dodecamer, EMD-25680), 7T4N (PhPINK1 D357A dimer, EMD-25681), 7T4L (with extended αC helix in chain B, EMD-25679), and 7T4K (wild-type phosphorylated PhPINK1 dimer with kinked αC helix in chain B, EMD-25678). We also deposited the map for wild-type phosphorylated PhPINK1 dimer prior to 3D variability analysis (EMD-25677). Uncropped versions of all gels and blots are provided in Supplementary Figure 1. Source data for Western blot quantification (Fig. 5f), melting curve measurements (Extended Data Fig. 3b) and quantifying imaging results (Extended Data Fig. 9g) are provided, the latter with corresponding Matlab script.

Field-specific reporting

Please select the one below that is the best fit for your research. If you are not sure, read the appropriate sections before making your selection.

☒ Life sciences ☐ Behavioural & social sciences ☐ Ecological, evolutionary & environmental sciences

For a reference copy of the document with all sections, see nature.com/documents/nr-reporting-summary-flat.pdf

Life sciences study design

All studies must disclose on these points even when the disclosure is negative.

Sample size	No sample size calculation was performed. The sample size (n) depicted in biochemical assays reflects the number of independently measured experiments. In cell biological experiments sample size (n) indicates the number of independent replicates performed. The number of technical and independent biological experiment is stated in each figure legend. Each experiment was successfully replicated at least three times.
Data exclusions	No data were excluded from analyses
Replication	Biochemical data including gel-based assays were reproduced with technical replicates and in independent experiments, with the number n of independent experiments stated in each figure legend. For gel-based assays, experiments were performed independently on different days. Uncropped gels are shown in Supplementary Information. A minimum of three repeats was performed for each experiment. All attempts at replication were successful.
Randomization	not applicable, as only a small number of samples were handled in each experiment.
Blinding	not applicable, as only a small number of samples were handled in each experiment.

Reporting for specific materials, systems and methods

We require information from authors about some types of materials, experimental systems and methods used in many studies. Here, indicate whether each material, system or method listed is relevant to your study. If you are not sure if a list item applies to your research, read the appropriate section before selecting a response.

Materials & experimental systems

n/a	Involved in the study	
☐	☒	Antibodies
☐	☒	Eukaryotic cell lines
☒	☐	Palaeontology and archaeology
☒	☐	Animals and other organisms
☒	☐	Human research participants
☒	☐	Clinical data
☒	☐	Dual use research of concern

Methods

n/a	Involved in the study	
☒	☐	ChIP-seq
☒	☐	Flow cytometry
☒	☐	MRI-based neuroimaging

Antibodies

Antibodies used	rabbit anti-PINK1 D8G3 (Cell Signaling Technology, #6946, lot 5); mouse anti-Parkin Prk8 (Cell Signaling Technology, #4211, lot 7), rabbit anti-phospho-ubiquitin (Ser65) (Millipore #ABS1513-I, lot 3117322); rabbit anti-TOM20 FL-145 (Santa Cruz Biotechnology

	sc-11415, lot D1613); goat anti-rabbit (SouthernBiotech, #4010-05, lot A4311-TF99D); goat anti-mouse (SouthernBiotech, #1030-05, lot E2518-Z929D); anti-tubulin hFAB rhodamine (Bio-Rad, #12004165, batch 64385864).
Validation	All primary antibodies have been validated for Western blotting in human cell lysates, by the manufacturer. Each primary antibody has featured in >50 publications as listed on the manufacturers websites.<br><br>PINK1 (https://www.cellsignal.com/products/primary-antibodies/pink1-d8g3-rabbit-mab/6946)<br>Parkin (https://www.cellsignal.com/products/primary-antibodies/parkin-prk8-mouse-mab/4211)<br>Phospho-ubiquitin (https://www.merckmillipore.com/AU/en/product/Anti-phospho-Ubiquitin-Ser65,MM_NF-ABS1513-I)<br>TOM20 (https://www.scbt.com/p/tom20-antibody-fl-145)<br>Tubulin (https://www.bio-rad.com/en-au/sku/12004165-hfab-rhodamine-anti-tubulin-primary-antibody-200-ul?ID=12004165)

Eukaryotic cell lines

Policy information about cell lines

Cell line source(s)	HeLa human PINK1 -/- cells (gift from Michael Lazarou, Monash University)
Authentication	Cell lines displayed expected cell morphologies and have been validated by the Garvan Molecular Genetics facility.
Mycoplasma contamination	Cells were tested negative for Mycoplasma. Cells were screened routinely for mycoplasma contamination using the MycoAlert Mycoplasma Detection Kit (Lonza) as per manufacturer's instructions.
Commonly misidentified lines<br>(See ICLAC register)	No misidentified cell lines have been used
