# Supplementary material for: Activation mechanism of PINK1
Source: Nature. 2021 Dec 21;602(7896):328–35. doi: 10.1038/s41586-021-04340-2 (PMC8828467; doi:10.1038/s41586-021-04340-2)
Supplement: Supplementary file 3 — Crystallographic and cryo-EM data. [file 41586_2021_4340_MOESM3_ESM.pdf]

Supplementary Table 1.

Crystallographic data collection and refinement statistics.

	***Ph*PINK1 D334A**
**Data collection**	
Space group	*P* 6_1
Cell dimensions	
a, *b*, *c* (Å)	116.12, 116.12, 70.57
α, β, γ (°)	90.00, 90.00, 120.00
Resolution (Å)	44.83 – 3.53 (3.87 – 3.53)
R_{merge} (within I+/I-)	0.360 (0.825)
$I / \sigma I$	4.2 (2.0)
Completeness (%)	99.9 (99.7)
Redundancy	5.7 (5.8)
**Refinement**	
Resolution (Å)	44.83 – 3.53
No. reflections	6751
R_{work} / R_{free}	0.2162 / 0.2495
No. atoms	
Protein	3071
Ligand/ion	0
Water	0
B-factors	
Protein	55.69
Ligand/ion	0
Water	0
R.m.s. deviations	
Bond lengths (Å)	0.002
Bond angles (°)	0.502

Values in parentheses are for highest-resolution shell.

Supplementary Table 2.

Cryo-EM data collection, refinement, and validation statistics

	*Ph*PINK1 D357A dodecamer (EMD-25680) (PDB 7T4M)	*Ph*PINK1 D357A dimer (EMD-25681) (PDB 7T4N)	Phospho-*Ph*PINK1 dimer prior to 3D variability analysis (EMD-25677)	Phospho-*Ph*PINK1 dimer "kinked αC" (EMD-25678) (PDB 7T4K)	Phospho-*Ph*PINK1 dimer "extended αC" (EMD-25679) (PDB 7T4L)
**Data collection and processing**					
Magnification	105,000×	105,000×	165,000×	165,000×	165,000×
Voltage (kV)	300	300	200	200	200
Electron exposure (e–/Å^2)	50	50	50	50	50
Defocus range (μm)	-0.4 to -1.9	-0.4 to -1.9	-0.6 to -2.0	-0.6 to -2.0	-0.6 to -2.0
Pixel size (Å)	0.83	0.83	0.78	0.78	0.78
Symmetry imposed	*D*3	*C*1	*C*1	*C*1	*C*1
Initial particle images (no.)	310,371	310,371	205,887	205,887	205,887
Final particle images (no.)	216,021	1,295,406	543,366	212,979	172,764
Map resolution (Å)	2.48	2.35	3.07	3.25	3.28
FSC threshold	0.143	0.143	0.143	0.143	0.143
Map resolution range (Å)	2.2-36.6	2.1-25.9	1.7-8.8	2.8-11.0	2.9-10.0
**Refinement**					
Initial model used (PDB code)	6EQI	6EQI		6EQI	6EQI
Model resolution (Å)	2.5	2.4		3.2	3.2
FSC threshold	0.143	0.143		0.143	0.143
Model resolution range (Å)					
Map sharpening *B* factor (Å^2)	-40	-35		-50	-50
Model composition					
Non-hydrogen atoms	39054	6476		6180	6062
Protein residues	4854	803		782	755
Ligands	0	0		0	0
B factors (Å^2)					
Protein	59.84	56.94		86.96	82.18
Ligand	0	0		0	0
R.m.s. deviations					
Bond lengths (Å)	0.002	0.002		0.005	0.004
Bond angles (°)	0.389	0.423		0.865	0.930
Validation					
MolProbity score	1.19	1.28		1.22	1.38
Clashscore	4.06	4.31		4.48	4.70
Poor rotamers (%)	0.55	1.25		0.00	0.00
Ramachandran plot					
Favored (%)	98.61	98.98		98.01	97.24
Allowed (%)	1.39	1.02		1.99	2.76
Disallowed (%)	0.00	0.00		0.00	0.00
